# Assessment of population infection with SARS-CoV-2 in Ontario, Canada, March to June 2020

**DOI:** 10.2807/1560-7917.ES.2021.26.50.2001559

**Published:** 2021-12-16

**Authors:** Shelly Bolotin, Vanessa Tran, Shelley L Deeks, Adriana Peci, Kevin A Brown, Sarah A Buchan, Katherene Ogbulafor, Tubani Ramoutar, Michelle Nguyen, Rakesh Thakkar, Reynato DelaCruz, Reem Mustfa, Jocelyn Maregmen, Orville Woods, Ted Krasna, Kirby Cronin, Selma Osman, Eugene Joh, Vanessa G Allen

**Affiliations:** 1 Public Health Ontario, Toronto, Ontario, Canada; 2 Dalla Lana School of Public Health, University of Toronto, Toronto, Ontario, Canada; 3 Department of Laboratory Medicine and Pathobiology, University of Toronto, Toronto, Ontario, Canada; 4 National Microbiology Laboratory, Public Health Agency of Canada, Winnipeg, Manitoba, Canada

**Keywords:** SARS-CoV-2, COVID-19, serology, seroprevalence

## Abstract

**Background:**

Serosurveys for SARS-CoV-2 aim to estimate the proportion of the population that has been infected.

**Aim:**

This observational study assesses the seroprevalence of SARS-CoV-2 antibodies in Ontario, Canada during the first pandemic wave.

**Methods:**

Using an orthogonal approach, we tested 8,902 residual specimens from the Public Health Ontario laboratory over three time periods during March–June 2020 and stratified results by age group, sex and region. We adjusted for antibody test sensitivity/specificity and compared with reported PCR-confirmed COVID-19 cases.

**Results:**

Adjusted seroprevalence was 0.5% (95% confidence interval (CI): 0.1–1.5) from 27 March–30 April, 1.5% (95% CI: 0.7–2.2) from 26–31 May, and 1.1% (95% CI: 0.8–1.3) from 5–30 June 2020. Adjusted estimates were highest in individuals aged ≥ 60 years in March–April (1.3%; 95% CI: 0.2–4.6), in those aged 20–59 years in May (2.1%; 95% CI: 0.8–3.4) and in those aged ≥ 60 years in June (1.6%; 95% CI: 1.1–2.1). Regional seroprevalence varied, and was highest for Toronto in March–April (0.9%; 95% CI: 0.1–3.1), for Toronto in May (3.2%; 95% CI: 1.0–5.3) and for Toronto (1.5%; 95% CI: 0.9–2.1) and Central East in June (1.5%; 95% CI: 1.0–2.0). We estimate that COVID-19 cases detected by PCR in Ontario underestimated SARS-CoV-2 infections by a factor of 4.9.

**Conclusions:**

Our results indicate low population seroprevalence in Ontario, suggesting that public health measures were effective at limiting the spread of SARS-CoV-2 during the first pandemic wave.

## Introduction

Severe acute respiratory syndrome coronavirus 2 (SARS-CoV-2), the causative agent of coronavirus disease (COVID-19), emerged as a novel pathogen in December 2019 [1] and has resulted in a global pandemic, with over 100 million cases and ca 2 million deaths reported by the end of January 2021 [2]. Canada’s first case of COVID-19 was reported in Toronto, Ontario on 25 January 2020 [3], when a traveller from Wuhan, China presented at the hospital with fever and cough [4]. By mid-March, in response to an increasing number of COVID-19 cases, the provincial Ontario government implemented physical distancing measures across Ontario, including limiting large gatherings and implementing school closures [5]. At the federal level, travel across the Canada–United States (US) border and internationally was restricted [6].

The first wave of the pandemic peaked in Ontario in mid-April, with declining case numbers through the summer of 2020, and a cumulative total by July 31, 2020 of nearly 40,000 cases and 2,800 deaths [7]. However, this number, which represents PCR-confirmed COVID-19 cases reported to Public Health Ontario (PHO), does not capture everyone in the population who has been infected, since not every infected individual is tested and reported [8]. There are several reasons for this, including a lack of clinical symptoms [9], individuals not presenting for assessment, limited availability of testing early in the pandemic, and other reasons why individuals may not seek or access laboratory testing.

The availability of serological testing for SARS-CoV-2 [10] enables the estimation of population infection over time through serosurveys [11]. Serosurveys are a valuable surveillance method to understand the spread of pathogens over time and to assess which groups in the population have been most affected. SARS-CoV-2 serosurveys provide an increased understanding of the true burden of infection, which will help determine the effectiveness of the pandemic response. Here we report the results of three cross-sectional serosurveys from Ontario during the first wave of the COVID-19 pandemic, performed using residual specimens from the PHO laboratory.

## Methods

### Study population and sampling strategy

We conducted a retrospective, repeated cross-sectional seroprevalence study to estimate SARS-CoV-2 infection in Ontario, Canada. We used residual sera, plasma and blood specimens left over after routine clinical testing at the PHO laboratory. The PHO laboratory is Ontario’s public health reference laboratory and is the largest public health laboratory in Canada, conducting over 6 million tests on a variety of sample types annually. The samples selected for this study were initially submitted for various diagnostic (March–June samples), and occupational and prenatal tests (June samples only), ensuring an adequate diversity of samples from all ages and Ontario regions. We excluded samples with missing information on age group, sex or geographical region of residence, samples without sufficient quantity, and those where the sample integrity was compromised. Specimens were de-identified before testing for SARS-CoV-2 antibodies.

**Figure 1:**
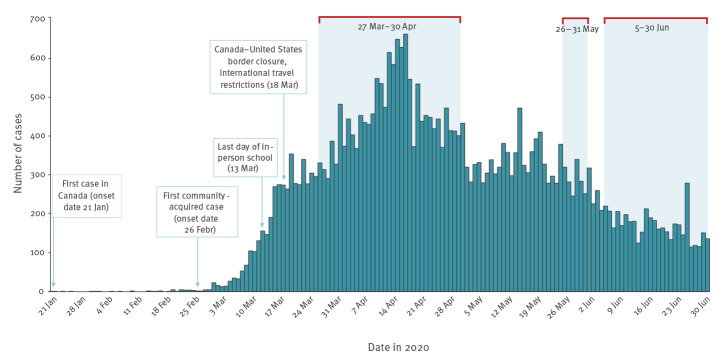
COVID-19 cases, dates of key public health measures, and serosurvey dates, Ontario, Canada, January–June 2020 (n = 35,217)

We tested residual specimens received in the PHO laboratory at three time points: between 27 March–30 April 2020 (the ‘March–April serosurvey’), 26–31 May 2020 (the ‘May serosurvey’) and 5–30 June, 2020 (the ‘June serosurvey’) (Figure 1). We aimed to test specimens that, as a group, were demographically representative of Ontario’s population. For the March–April and May serosurveys, we used broad age groups (0–19, 20–59 and ≥ 60 years) and geographical criteria (Northern, Eastern, Western and Toronto regions) to select specimens for testing on account of a scarcity of samples in March–April, when most healthcare services in Ontario were unavailable or limited, and a shorter selection period of only 6 days in May, because of operational issues in the laboratory. For the June serosurvey, we aimed for proportional representation by age group (0–4, 5–9, 10–19, 20–29, 30–39, 40–49, 50–59, 60–69, 70–79, ≥ 80 years) and sex, as per The Unity Studies: World Health Organization Sero-epidemiological Investigations protocols [12], and geographical balance (Northern, Eastern, Central East, Toronto, South West, Central West regions). To ensure that serosurvey results contribute to the Canadian pandemic response in a timely manner, some of the results presented here were published on the Public Health Ontario website [13].

COVID-19: coronavirus disease.

Cases of COVID-19 were sourced from the Public Health Ontario COVID-19 Data Tool, and are reported by episode date, which is an estimate of the symptom onset date. Dates of key public health measures were reported in various sources [33-37]. The three serosurvey periods are represented by red brackets and light blue shading.

### Sample size calculations

Our sample size calculations, performed using Power Analysis and Sample Size (PASS) software version 15 (NCSS, LLC, Kaysville, Utah, US), indicated that for 1,000 samples, i.e. the approximate size of our March–April and May surveys and the upper limit sample size per 10-year age group for our June survey, the 95% confidence intervals (CI) of our estimates would be approximately ± 1.5% if seroprevalence was 5% and ± 2.0% if seroprevalence was 10%.

### Serological testing

We tested specimens using an orthogonal approach, where specimens were analysed using two independent tests in sequential approach [14]. All samples were first tested using the Abbott Architect SARS-CoV-2 IgG assay (Abbott Laboratories, Abbott Park, Illinois, US), which detects anti-nucleocapsid antibodies. Samples that were positive with the Architect SARS-CoV-2 IgG assay were then tested using the VITROS anti-SARS-CoV-2 IgG assay, which detects anti-spike antibodies (Ortho-Clinical Diagnostics, Raritan, New Jersey, US). These assays were validated in-house with the same set of specimens and were each found to have 92.3% (95% CI: 81.5–97.9) sensitivity for specimens collected > 14 days from symptom onset or from date of PCR specimen collection, and 100.0% (95% CI: 96.4–100.0) specificity. Only specimens that tested positive by both assays were considered positive, otherwise they were considered negative.

### Epidemiological analysis

We conducted a descriptive epidemiological analysis to estimate the proportion of samples with positive results for SARS-CoV-2 antibodies for each serosurvey, by age group and sex, and in each region in Ontario. We calculated 95% CIs based on the Wald method when the numerator was 5 or more, and based on the Clopper-Pearson method when the numerator was less than 5. We then sequentially adjusted prevalence estimates and 95% CIs to account for differences between the sample and population structure of Ontario, as well as the sensitivity and specificity of the orthogonal testing approach.

First, we developed and applied post-stratification weights derived from Ontario population projection data for 2020, which were sourced from the Ontario Ministry of Health, IntelliHEALTH Ontario [15]. Weights were based on age group (0–19, 20–59, and ≥ 60 years), sex, and region (Toronto, Central East and Central West vs Northern, Eastern, and South West), and were equal to the inverse of the probability of selection. Using the weighting-adjusted prevalence estimates, we then adjusted for test characteristics of the orthogonal approach, i.e. combined sensitivity of 90.4% (95% CI: 79.0–96.8); combined specificity of 100% (95% CI: 96.4–100.0) using the following formula:

prevalence = (test prevalence + specificity − 1)/(sensitivity + specificity − 1).

All analyses were conducted using R version 3.6.3 (R Foundation, Vienna, Austria).

### Sensitivity analysis

We conducted a sensitivity analysis to explore the impact of uncertainty in our estimates of the sensitivity and specificity of the SARS-CoV-2 IgG assays used in our orthogonal testing approach. Specifically, we used plausible estimates of sensitivity and specificity to produce low (high sensitivity and low specificity), medium, and high (low sensitivity and high specificity) estimates of seroprevalence in our population. First, we used the 75^th^ percentile of our estimated sensitivity (93.5%) and the manufacturer-reported specificity (99.6%) for the first test used in our orthogonal approach (Abbott Architect assay) [16]. Second, we used the point estimate of sensitivity (90.4%) and used a specificity of 99.8%, which was based on the manufacturer estimate, as well as an assumption of 50% specificity for the second test (Ortho-Diagnostics VITROS assay). Third, we used the 25^th^ percentile of our estimate sensitivity (86.1%) with the point estimate of specificity (100%).

### Estimating the number of infections in Ontario and COVID-19 under-reporting

To estimate the number of SARS-CoV-2 infections in the Ontario population, we multiplied the overall adjusted seroprevalence for the June survey by the population of Ontario [15]. To estimate the degree of under-reporting of cases, we compared the resulting estimate of infection to the number of PCR-confirmed COVID-19 cases reported in Ontario 2 weeks before the end of the June serosurvey. This was to account for the fact that antibody responses can take up to 2 weeks to be generated, and a small proportion of cases do not seroconvert [17]. The range in under-reporting was calculated using the upper and lower limits of the 95% CI estimates of the adjusted seroprevalence values.

### Ethical statement

Our study received ethical approval from the PHO Ethics Review Board (approval numbers 2020-013 and 2020-023). We engaged the Ontario COVID-19 Ethics Table [18] with additional consultation.

## Results

### Seroprevalence findings

We tested a total of 8,902 samples for IgG antibodies to SARS-CoV-2. Of these, 827 were submitted to our laboratory in March–April 2020; 1,061 were submitted in May 2020; and 7,014 were submitted in June 2020. The proportion of specimens from males ranged from 40.7% (337/827) in March–April to 49.5% (525/1,061) in May, while those from females ranged from 50.5% (536/1,061) in May to 59.3% (490/827) in March–April (Table 1). The proportion of specimens by age group ranged from 13.9% (978/7,014) in June to 22.0% (182/827) in March–April from individuals 0–19 years; 49.1% (521/1,061) in May to 60.8% (503/827) in March–April from individuals age 20–59 years; and 17.2% (142/827) in March–April to 30.3% (322/1,061) in May from individuals age ≥ 60 years (Table 1). By region, the proportion of specimens ranged from 2.7% (29/1,061) from Eastern Ontario in May to 37.6% (399/1,061) from Central East Ontario, also for the May survey. For context, these regions contribute 13.0% and 30.1% of Ontario’s population of 14.8 million, respectively [15].

**Table 1:** Demographic characteristics of the study population by SARS-CoV-2 serosurvey collection period, Ontario, Canada, March–June 2020 (n = 8,902)

Characteristics	Collection period and number of specimens						Distribution in the Ontario population^a^ (%)
	27 March–30 April (n = 827)		26–31 May (n = 1,061)		5–30 June (n = 7,014)		
	n	%	n	%	n	%	
Sex							
Male	337	40.7	525	49.5	3,423	48.8	49.2
Female	490	59.3	536	50.5	3,591	51.2	50.8
Age group (years)							
0–19	182	22.0	218	20.5	978	13.9	21.1
20–59	503	60.8	521	49.1	3,996	57.0	54.5
≥ 60	142	17.2	322	30.3	2,040	29.1	24.4
Region							
Northern	238	28.8	74	7.0	422	6.0	5.4
Eastern	47	5.7	29	2.7	627	8.9	13.0
Central East	205	24.8	399	37.6	2,446	34.9	30.1
Toronto	259	31.3	275	25.9	1,837	26.2	21.0
South West	30	3.6	93	8.8	446	6.4	11.4
Central West	48	5.8	191	18.0	1,236	17.6	19.2

SARS-CoV-2: severe acute respiratory syndrome coronavirus 2.

^a^ A population of 14.8 million, sourced from the Ontario Ministry of Health, was used to estimate the per cent distribution for each region [15].

Using an orthogonal testing approach, we first tested all specimens using the Abbott Architect assay. Of 8,902 specimens, 1.5% (n = 131) were positive using the Abbott assay (Table 2). The positive specimens were then retested using the Ortho-Diagnostics VITROS assay.

**Table 2:** Orthogonal testing results with two SARS-CoV-2 IgG assays, Ontario, Canada, March–June 2020 (n = 8,902)

Anti-SARS-CoV-2 IgG assay		Abbott Architect assay		Total	
		Positive (n)	Negative (n)	n	%
Ortho-Diagnostics VITROS assay	Positive (n)	97	0	97	1.1
	Negative (n)	34	0	34	0.4
	Not retested (n)	0	8,771	8,771	98.5
	Total (n)	131	8,771	8,902	100

SARS-CoV-2: severe acute respiratory syndrome coronavirus 2.

The tests used were the Abbott Architect SARS-CoV-2 IgG assay (Abbott Laboratories, Abbott Park, Illinois, US), which detects anti-nucleocapsid antibodies, and the VITROS anti-SARS-CoV-2 IgG assay (Ortho-Clinical Diagnostics, Raritan, New Jersey, US), which detects anti-spike antibodies.

**Figure 2:**
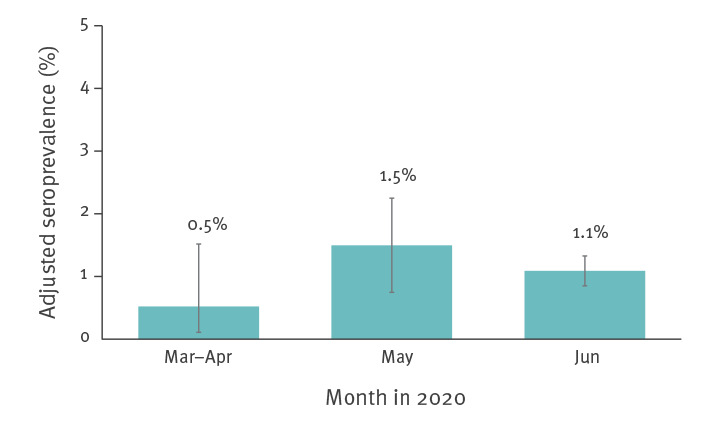
Adjusted SARS-CoV-2 IgG antibody seroprevalence by serosurvey collection period, Ontario, Canada, March–June 2020 (n = 8,902)

Of these, 74.0% (97/131) were positive. The total number of specimens that were positive using both tests was 0.4% (3/827; 95% CI: 0.07–1.1) for March–April, 1.4% (15/1,061; 95% CI: 0.7–2.1) for May and 1.1% (79/7,014; 95% CI: 0.9–1.4) for June. Adjusted for population weighting and serology test characteristics, seroprevalence was 0.5% (95% CI: 0.1–1.5) for March–April, 1.5% (95% CI: 0.7–2.2) in May, and 1.1% (95% CI: 0.8–1.3) in June (Figure 2 and Table 3). From here forward, we will report adjusted seroprevalence estimates only.

COVID-19: coronavirus disease. SARS-CoV-2: severe acute respiratory syndrome coronavirus 2.

The three serosurveys were performed between the following dates: 27 March–30 April 2020 (March–April survey), 26–31 May 2020 (May survey) and 5–30 June 2020 (June survey). Seroprevalence estimates were adjusted for population weighting and serology test characteristics. The solid bars indicate adjusted seroprevalence and the error bars indicate 95% confidence intervals.

**Table 3:** Proportion of SARS-CoV-2 IgG antibody-positive samples and adjusted seroprevalence overall, by age group, sex and geographical region, Ontario, Canada, 27 March–30 June 2020 (n = 8,902)

Characteristics	Collection period														
	27 March–30 April					26–31 May					5–30 June				
	Antibody-positive samples			Adjusted seroprevalence		Antibody-positive samples			Adjusted seroprevalence		Antibody-positive samples			Adjusted seroprevalence	
	n/N	%	95% CI	%	95% CI	n/N	%	95% CI	%	95% CI	n/N	%	95% CI	%	95% CI
Overall	3/827	0.4	0.07–1.1	0.5	0.1–1.5	15/1,061	1.4	0.7–2.1	1.5	0.7–2.2	79/7,014	1.1	0.9–1.4	1.1	0.8–1.3
Sex															
Female	0/490	0.0	0.0–0.8	0.0	0.0–0.8	9/536	1.7	0.6–2.8	1.8	0.6–3.0	34/3,591	0.9	0.6–1.3	1.0	0.7–1.3
Male	3/337	0.9	0.2–2.6	1.1	0.2–3.1	6/525	1.1	0.2–2.1	1.1	0.2–2.1	45/3,423	1.3	0.9–1.7	1.2	0.9–1.6
Age group (years)															
0–19	0/182	0.0	0.0–2.0	0.0	0.0–2.2	2/218	0.9	0.1–3.3	0.7	0.08–2.4	9/978	0.9	0.3–1.5	0.8	0.3–1.4
20–59	1/503	0.2	0.005–1.1	0.4	0.01–2.1	10/521	1.9	0.7–3.1	2.1	0.8–3.4	36/3,996	0.9	0.6–1.2	1.0	0.7–1.3
≥ 60	2/142	1.4	0.2–5.0	1.3	0.2–4.6	3/322	0.9	0.2–2.7	0.8	0.2–2.4	34/2,040	1.7	1.1–2.2	1.6	1.1–2.1
Region															
Northern	0/238	0.0	0.0–1.5	0.0	0.0–1.7	1/74	1.4	0.03–7.3	1.3	0.03–7.2	1/422	0.2	0.006–1.3	0.3	0.009–1.9
Eastern	0/47	0.0	0.0–7.5	0.0	0.0–8.4	0/29	0.0	0.0–11.9	0.0	0.0–13.2	2/627	0.3	0.04–1.1	0.3	0.04–1.1
Central East	1/205	0.5	0.01–2.7	0.8	0.02–4.2	2/399	0.5	0.06–1.8	0.7	0.08–2.4	38/2,446	1.6	1.1–2.0	1.5	1.0–2.0
Toronto	2/259	0.8	0.1–2.8	0.9	0.1–3.1	8/275	2.9	0.9–4.9	3.2	1.0–5.3	26/1,837	1.4	0.9–2.0	1.5	0.9–2.1
South West	0/30	0.0	0.0–11.6	0.0	0.0–12.8	1/93	1.1	0.03–5.8	0.9	0.02–5.1	2/446	0.4	0.05–1.6	0.4	0.05–1.5
Central West	0/48	0.0	0.0–7.4	0.0	0.0–8.2	3/191	1.6	0.3–4.5	1.8	0.4–5.2	10/1,236	0.8	0.3–1.3	1.1	0.4–1.7

CI: confidence interval; SARS-CoV-2: severe acute respiratory syndrome coronavirus 2.

Adjusted seroprevalence estimates for the sub-groups varied by serosurvey period (Table 3). Adjusted seroprevalence by sex did not vary substantially in any time period nor between the sexes at any time period. Seroprevalence was higher in males compared with females in March–April, when seropositivity was 1.1% (95% CI: 0.2–3.1) in males compared with 0.0% (95% CI: 0.0–0.8) in females, and in June, when seropositivity was 1.2% (95% CI: 0.9–1.6) in males compared with 1.0% (95% CI: 0.7–1.3) in females. Conversely, in May, seroprevalence was 1.1% (95% CI: 0.2–2.1) and 1.8% (0.6–3.0) in males and females, respectively.

Similarly, although there were no substantial differences by age group across collection periods, adjusted seroprevalence was highest in specimens from individuals aged ≥ 60 years in March–April, at 1.3% (95% CI: 0.2–4.6), in specimens from individuals aged 20–59 years in May at 2.1% (95% CI: 0.8–3.4), and in specimens from individuals aged ≥ 60 years in June at 1.6% (95% CI: 1.1–2.1) (Table 3). Specimens from individuals age 0–19 years had the lowest seroprevalence of all age groups in all three time periods, at 0.0% (95% CI: 0.0–2.2) for March–April, 0.7% (95% CI: 0.08–2.4) for May and 0.8% (95% CI: 0.3–1.4) for June.

**Figure 3:**
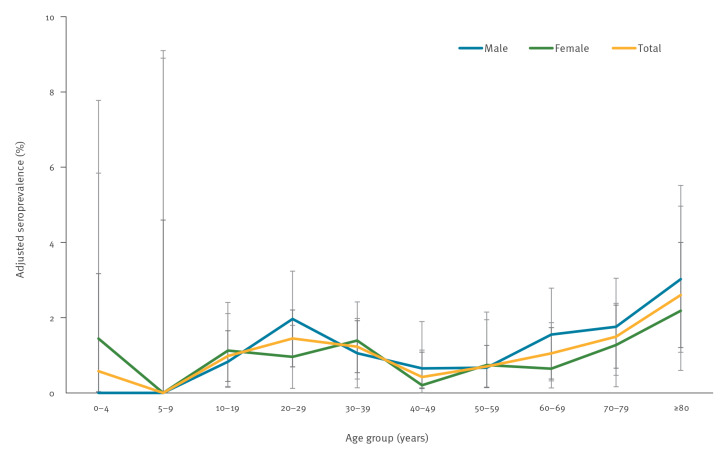
Adjusted SARS-CoV-2 IgG antibody seroprevalence by age group and sex, Ontario, Canada, 5–30 June 2020 (n = 7,014)

The larger sample size for the June serosurvey allowed us to analyse seroprevalence by narrower age groups (Figure 3). Similar to the March–April and May serosurveys, seroprevalence was generally lower in children aged 19 years and under, with adjusted estimates of 0.6% (95% CI: 0.01–3.2) for children age 0–4 years, 0.0% (95% CI: 0.0–4.6) for children aged 5–9 years and 1.0% (95% CI: 0.3–1.7) in older children and teens aged 10–19 years. In adults, adjusted seroprevalence was highest in older individuals, at 1.5% (95% CI: 0.7–2.3) in those age 70–79 and 2.6% (95% CI: 1.2–4.0) in those ≥ 80 years. While in some age groups, males had higher seroprevalence and in others, females had higher seroprevalence; all CIs overlapped.

SARS-CoV-2: severe acute respiratory syndrome coronavirus 2.

Coloured lines represent the mean adjusted seroprevalence for males, females, and the total. Error bars represent 95% confidence intervals for each group.

**Figure 4:**
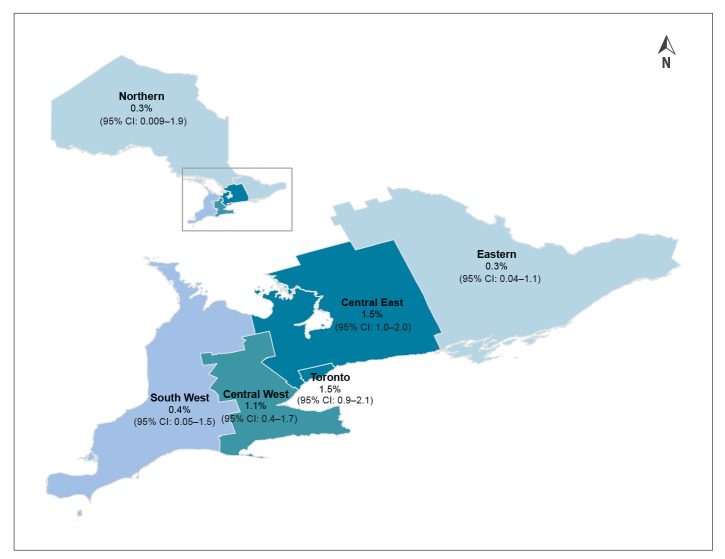
Adjusted SARS-CoV-2 IgG antibody seroprevalence by region, Ontario, Canada, 5–30 June 2020 (n = 7,014)^a^

CI: confidence interval; SARS-CoV-2: severe acute respiratory syndrome coronavirus 2.

^a^ Population in each region [15]: Northern (n = 796,974); Eastern (n = 1,926,380); Central East (n = 4,480,654); Toronto (n = 3,120,358); Central West (n = 2,849,288); South West (n = 1,690,774).

Regionally, seroprevalence was highest in Toronto and surrounding areas (Central East and Central West) for all surveys (Table 3). In March–April, all three positive specimens of 827 originated from individuals residing in Toronto or Central East Ontario, which includes suburban public health units immediately surrounding Toronto (Figure 4). Adjusted seroprevalence for the March–April survey was 0.9% (95% CI: 0.1–3.1) for Toronto and 0.8% (95% CI: 0.02–4.2) for Central East Ontario. In May, the majority of positive specimens (10/15; 66.7%) also originated from the Toronto or Central East regions, with an adjusted seroprevalence of 3.2% (95% CI: 1.0–5.3) and 0.7% (95% CI: 0.08–2.4), respectively. Central West Ontario also had notable seroprevalence, at 1.8% (95% CI: 0.4–5.2). June followed a similar pattern, with 74/79 (93.7%) positive specimens originating from Toronto, Central East and Central West, with adjusted seroprevalence of 1.5% (95% CI: 0.9–2.1), 1.5% (95% CI: 1.0–2.0) and 1.1% (95% CI: 0.4–1.7), respectively. The Northern, Eastern and South West regions had the lowest number of positive specimens throughout, with no positive specimens in March–April, and 2 or fewer positive specimens from each region in May and June. However, the smaller sample sizes from these regions overall impact both the stability and precision of the seroprevalence estimates somewhat, especially in May. In June, these three regions had the lowest adjusted seroprevalence, at 0.3% (95% CI: 0.009–1.9) in Northern, 0.3% (95% CI: 0.04–1.1) in Eastern and 0.4% (95% CI: 0.05–1.5) in South West Ontario (Figure 4).

### Sensitivity analyses

Sensitivity analyses examining alternative plausible test sensitivities and specificities suggested that SARS-CoV-2 seroprevalence in June may have been as low as 0.6% (95% CI: 0.4–0.9) if test sensitivity was higher (93.5%) and specificity was lower (99.6%) or similar to the original estimate at 1.1% (95% CI: 0.9–1.4) if test sensitivity was lower (86.1%) and specificity was 100% (Table 4).

**Table 4:** Sensitivity analysis examining alternative plausible sensitivities and specificities for SARS-CoV-2 IgG antibody tests, Ontario, Canada, 27 March–30 June 2020 (n = 8,902)

Adjustment for anti-SARS-CoV-2 antibody test characteristics			Collection period and seroprevalence estimates					
			27 March–30 April		26–31 May		5–30 June	
SARS-CoV-2 antibody seroprevalence estimate	Sensitivity	Specificity						
	%	%	%	95% CI	%	95% CI	%	95% CI
Main	90.4^a^	100^a^	0.5	0.1–1.5	1.5	0.7–2.2	1.1	0.8–1.3
Low	93.5	99.6	0.08	0.0–1.0	1.0	0.3–1.8	0.6	0.4–0.9
Medium	90.4	99.8	0.3	0.0–1.3	1.3	0.5–2.0	0.9	0.6–1.1
High	86.1	100	0.6	0.1–1.6	1.6	0.8–2.4	1.1	0.9–1.4

CI: confidence interval; SARS-CoV-2: severe acute respiratory syndrome coronavirus 2.

^a^ Main analysis.

The tests used were the Abbott Architect SARS-CoV-2 IgG assay (Abbott Laboratories, Abbott Park, Illinois, US), which detects anti-nucleocapsid antibodies, and the VITROS anti-SARS-CoV-2 IgG assay (Ortho-Clinical Diagnostics, Raritan, New Jersey, US), which detects anti-spike antibodies.

### Estimating the burden of COVID-19 in Ontario

SARS-CoV-2 seroprevalence estimates can be used to approximate the number of individuals who have been infected. This number can then be compared to the reported number of COVID-19 cases detected through PCR testing, which is currently the gold standard used for the laboratory diagnosis of COVID-19. Extrapolating the adjusted June seroprevalence estimate of 1.1% to the Ontario population, we estimate that ca 162,000 individuals of 14.8 million Ontario residents were likely infected with COVID-19. This is compared with 32,744 cases reported by 16 June 2020 [19]. This suggests that cases detected by PCR underestimate COVID-19 infections by a factor of 4.9 (range: 3.9–6.0). Since not all individuals seroconvert upon infection with SARS-CoV-2, it is also possible that seroprevalence estimates underestimate the burden of disease.

## Discussion

In this study, we present SARS-CoV-2 seroprevalence estimates for Ontario, Canada during the first wave of the COVID-19 pandemic. Ontario is Canada’s largest province, with an estimated population of 14.8 million residents, comprising 39% of the Canadian population [20]. The overall seroprevalence trends for Ontario are concordant with reported PCR-confirmed COVID-19 cases [7]. Similar to the highest rate of reported COVID-19 cases confirmed by PCR, the highest adjusted seroprevalence was in individuals aged 80 years and older. Other age groups with notable seroprevalence estimates were also consistent with age groups that showed elevated case rates by PCR, including individuals aged 60–79 years and 20–39 years [7]. Paediatric age groups had the lowest seroprevalence estimates, also concordant with reported Ontario case rates. By region, both the highest seroprevalence estimates and the highest case rates were in Toronto and Central East Ontario, with Central West Ontario having the next highest rates and seroprevalence estimates [21]. Northern, Eastern and South West Ontario’s seroprevalence were the lowest of all regions. The seroprevalence estimate for Northern Ontario mirrored case rates in this region at the time of publication, with the lowest rate of all regions reported. However, case rates in Eastern and South West Ontario were markedly higher than those in the North, which was not reflected in our seroprevalence findings. The small numbers of positive specimens, which resulted in large CIs, indicate that estimates for these regions are less precise than for other regions.

One marked difference in seroprevalence estimates compared with COVID-19 case rates was the estimated number of infections, which we extrapolated to be nearly five times higher compared with reported cases. However, it is well-known that COVID-19 is substantially under-reported [8]. Our finding of nearly a fivefold under-reporting is in line with results using another methodology in Ontario, which back-calculates infections from the number of COVID-19 deaths reported to the public health system. Using this method, the number of COVID-19 cases in mid-May in Ontario was found to be nearly fourfold higher than those reported through case detection [22].

Our findings are comparable to those from other Canadian seroprevalence studies. Our Ontario estimate is very similar to the reported Ontario SARS-CoV-2 seroprevalence estimate (0.96%) from the Canadian Blood Services seroprevalence study for May and June 2020 [23], and slightly higher but comparable to that reported from the Lower Mainland region of British Columbia in May (0.55%), where COVID-19 incidence was lower and case numbers decreased earlier than Ontario [24]. Our estimates are also comparable to those from other jurisdictions that used residual sera specimens, including Western Washington State and the San Francisco Bay area in the US in April 2020 (adjusted seroprevalence of 1.1% and 1.0%, respectively) [25]. Serosurvey estimates from European countries like Norway and Germany, which implemented public health restrictions on a similar timeline to Ontario, were also comparable, i.e. 1.0% in Norway in April–May, and 0.9% in Germany in March–June [26,27]. Our estimates were lower than in New York City, US in April 2020 (19.3%), which was one of the first US jurisdictions to record a high morbidity and mortality, with ca 221,000 COVID-19 cases and over 20,000 probable and confirmed deaths by the end of July [28,29].

The large sample informing this serosurvey and our choice of serology assays and orthogonal testing approach are strengths of our study. We used two laboratory-based anti-SARS-CoV-2 IgG assays in an orthogonal algorithm rather than lateral flow assays, which reduced the probability of false positives and provided a more robust estimate when the prevalence was low. Our previous serological test validation estimated a high orthogonal combined test sensitivity of 90.4% and specificity of 100%. Because of the imperfect characteristics of current SARS-CoV-2 antibody tests, and the fact that test result interpretation of residual specimens is difficult without accompanying clinical and epidemiological data, it is essential that test methods are as accurate as possible [14]. Furthermore, the low prevalence of anti-SARS-CoV-2 antibodies in the Ontario population could result in a low positive predictive value, meaning that specificity in particular needs to be as high as possible, making an orthogonal testing approach critical [30]. Despite the fact that our laboratory validation demonstrated 100% specificity of our orthogonal testing approach, it is possible that specificity may be lower, which we addressed with various sensitivity analyses.

There are limitations associated with using residual specimens for serosurveillance. Epidemiological data available with residual specimens, which were initially collected for other purposes, are usually limited. For our study, having data elements related to healthcare utilisation, race and socioeconomic status would have been helpful to enable us to explore the correlation between these factors and antibody status, and to understand whether our specimens were representative by these characteristics. In addition, residual specimens may not represent healthy individuals, who may not routinely have blood drawn. This may particularly be the case for our first two serosurveys in March–April and May, during which access to laboratory testing was limited because of a provincial lockdown. However, this limitation may have been somewhat mitigated in our June study sample which, in addition to specimens submitted for diagnostic testing, includes specimens submitted for prenatal and occupational testing, i.e. assessment of healthcare worker immunity, drawing from healthy populations. One exception is paediatric specimens, as children rarely have blood drawn, and those who do may be more likely to have underlying conditions and therefore not representative of the general paediatric population. A second exception includes vulnerable populations, e.g. migrant workers, people who are experiencing homelessness or are underhoused, who may not be captured because of differences in how they access routine clinical services and laboratory testing. Since some vulnerable populations experienced a higher incidence of COVID-19 than the general population, it is possible that our seroprevalence study may underestimate the burden of infection. Our sample selection approach is specimen-based and not person-based, and it is therefore possible that more than one specimen was tested per individual. Lastly, a general limitation of serosurveillance studies for COVID-19 is that they may underestimate the burden of infection, since it takes up to 2 weeks to generate an antibody response to COVID-19. A small proportion of infected individuals do not seroconvert [17], and some studies have shown that antibody responses after infection decline over time [17,31].

## Conclusions

SARS-CoV-2 seroprevalence estimates for Ontario during the first pandemic wave, from March to June 2020, suggest that public health measures, which included physical distancing, school closures and lockdown, as well as rapid expansion of laboratory testing and case and contact management [32], were effective in limiting the spread of SARS-CoV-2 throughout the province. These estimates have also indicated that PCR-confirmed COVID-19 cases under-reported the true burden of disease in Ontario nearly fivefold. However, the serological findings also confirmed epidemiological findings from reported data on areas with greatest burden of disease. They demonstrate the utility of serosurveys as a valuable surveillance data stream to monitor the proportion of the population, as well as sub-groups, which have been infected with SARS-CoV-2. These data from the early stages of the pandemic will serve as baseline for future comparisons.
